# In Vivo Response of Growth Plate to Biodegradable Mg-Ca-Zn Alloys Depending on the Surface Modification

**DOI:** 10.3390/ijms20153761

**Published:** 2019-08-01

**Authors:** Mi Hyun Song, Won Joon Yoo, Tae-Joon Cho, Yong Koo Park, Wang-Jae Lee, In Ho Choi

**Affiliations:** 1 Department of Orthopaedic Surgery and Institute for Rare Diseases, Korea University Medical Center Guro Hospital, 148 Gurodong-ro, Guro-gu, Seoul 08308, Korea; 2 Division of Pediatric Orthopaedics, Seoul National University Children’s Hospital, 101 Daehak-ro, Jongno-gu, Seoul 03080, Korea; 3 Department of Pathology, Kyung Hee University Medical Center, 23 Kyungheedae-ro, Dongdaemun-gu, Seoul 02447, Korea; 4 Department of Anatomy and Tumor Immunity Medical Research Center, 101 Daehak-ro, Jongno-gu, Seoul National University College of Medicine, Seoul 03080, Korea; 5 Department of Orthopaedic Surgery, Chung-Ang University Medical Center, 102 Heukseok-ro, Dongjak-gu, Seoul 06973, Korea

**Keywords:** biodegradable, Mg-Ca-Zn alloy, plasma electrolyte oxidation, growth plate

## Abstract

Because Mg-Ca-Zn alloys are biodegradable and obviate secondary implant removal, they are especially beneficial for pediatric patients. We examined the degradation performance of Mg-Ca-Zn alloys depending on the surface modification and investigated the in vivo effects on the growth plate in a skeletally immature rabbit model. Either plasma electrolyte oxidation (PEO)-coated (*n* = 18) or non-coated (*n* = 18) Mg-Ca-Zn alloy was inserted at the distal femoral physis. We measured the degradation performance and femoral segment lengths using micro-CT. In addition, we analyzed the histomorphometric and histopathologic characteristics of the growth plate. Although there were no acute, chronic inflammatory reactions in either group, they differed significantly in the tissue reactions to their degradation performance and physeal responses. Compared to non-coated alloys, PEO-coated alloys degraded significantly slowly with diminished hydrogen gas formation. Depending on the degradation rate, large bone bridge formation and premature physeal arrest occurred primarily in the non-coated group, whereas only a small-sized bone bridge formed in the PEO-coated group. This difference ultimately led to significant shortening of the femoral segment in the non-coated group. This study suggests that optimal degradation could be achieved with PEO-coated Mg-Ca-Zn alloys, making them promising and safe biodegradable materials with no growth plate damage.

## 1. Introduction

Mg-based materials are promising in the orthopaedic field because of their various advantages, such as biodegradability [1,2,3] and biocompatibility. Hence they do not induce systemic inflammatory reaction [4,5]. In addition, the mechanical properties of these materials closely resemble those of cortical bone [5,6,7], which leads to a reduced stress shielding reaction as compared to what is observed with conventional materials used in orthopaedic implants. Therefore, Mg-based materials can provide enough stability for achieving union at the osteotomy/fracture site and also reduce the burdens of secondary implant removal, which benefits the patients [7]. In particular, the use of these materials would be greatly welcomed in pediatric patients who have a phobia of anesthesia and repeated surgeries.

Despite the sanguine views, there are several points to be considered before using these materials in the pediatric orthopaedic field. First is the selection of appropriate Mg-based material which can minimize its peculiar drawback. The main drawback of these materials is the formation of hydrogen gas and aqueous solutions during their rapid degradation [3,7,8,9], which leads to further implant degradation [10,11]. Accordingly, various attempts have been made to produce Mg alloys and modify their surface to slow the degradation rate and regulate the by-product formation [2,7,8,12,13,14]. In this process, some Mg alloys even contain aluminum or heavy rare earth elements [1,10,12,14,15]. However, these elements could exhibit toxic effects on the adjacent and distant tissues [10,15,16,17]. Thus, we used Mg-Ca-Zn alloy instead, because calcium (Ca) and zinc (Zn) are relatively biocompatible. The corrosion resistance and mechanical properties of Mg-Ca-Zn alloys are also better compared to those of pure Mg [5,11,13,18]. Moreover, before using Mg-Ca-Zn alloys in the case of pediatric patients, it is necessary to ensure that the alloy does not damage the growth plate as it may affect the normal growth of the patients carrying them. Only one previous study investigated the effect of Mg alloy on the growth plate [15]. While they demonstrated that biodegradable Mg alloys (containing yttrium) did not adversely affect the growth plate [15], they concluded that Mg alloys containing rare earth elements did not recommend using in skeletal immature patients. Except that, there has been no study that investigated the effects of biodegradable Mg alloys on the growth plate. The aim of this study was to examine the degradation performance of Mg-Ca-Zn alloys depending on the surface modification, and to investigate the in vivo effects of Mg-Ca-Zn alloys on the growth plate in a rabbit model. To the best of our knowledge, this study is the first investigation of the in vivo response of growth plate to biodegradable Mg-Ca-Zn alloys.

## 2. Results

### 2.1. Degradation Performance

**Figure 1:**
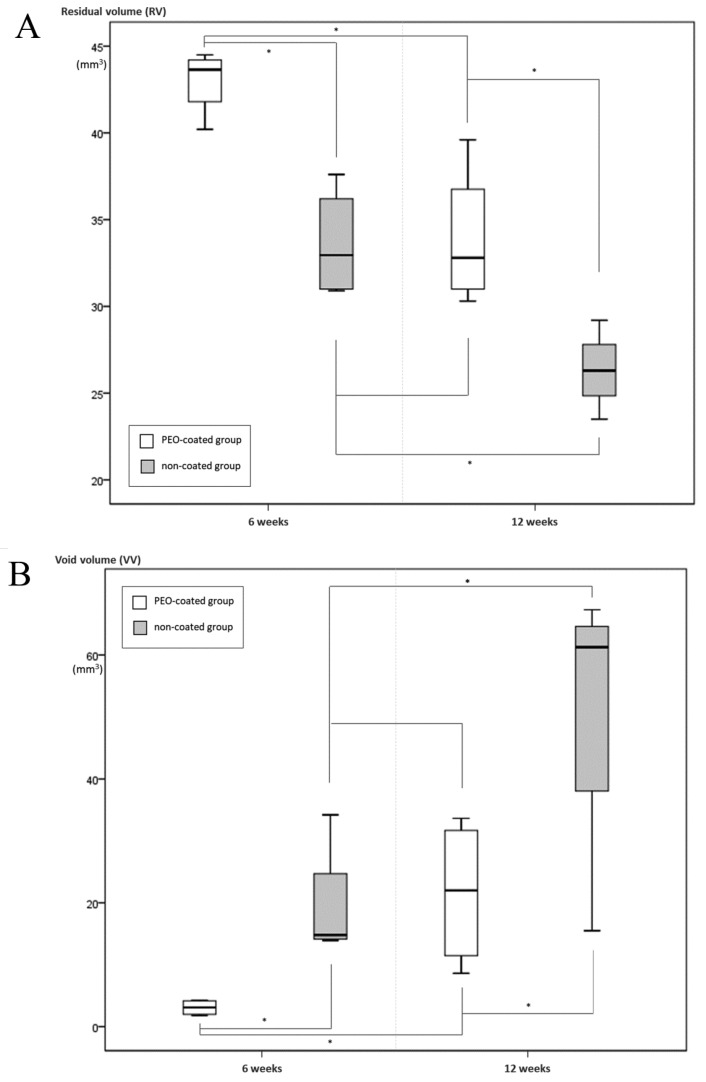
Degradation performance of both PEO-coated and non-coated Mg-Ca-Zn alloys. (**A**) The residual volumes of the PEO-coated alloys were significantly larger than those of the non-coated alloys at every time point. The initial Mg alloy volume was 44.87 mm^3^. Note the asterisk (*) indicates that the p-value was under 0.05. (**B**) The void volumes of PEO-coated alloys were smaller than those of non-coated alloys at every time point. Note the asterisk (*) indicates that the *p*-value was under 0.05.

The residual volume (RV) of the plasma electrolyte oxidation (PEO)-coated Mg-Ca-Zn alloys was significantly larger than those of non-coated alloys at every time point (Figure 1A). The degradation rates of the PEO-coated alloys were significantly lower than those of the non-coated alloys (Table 1). The void volumes of the PEO-coated alloys were noticeably smaller than those of the non-coated alloys at every time point (Figure 1B). These results suggest that the PEO-coated alloys degraded significantly more slowly than the non-coated ones and that they created significantly less hydrogen gas than did the non-coated alloys throughout the follow-up period.

### 2.2. Overall Tissue Reactions 

**Figure 2:**
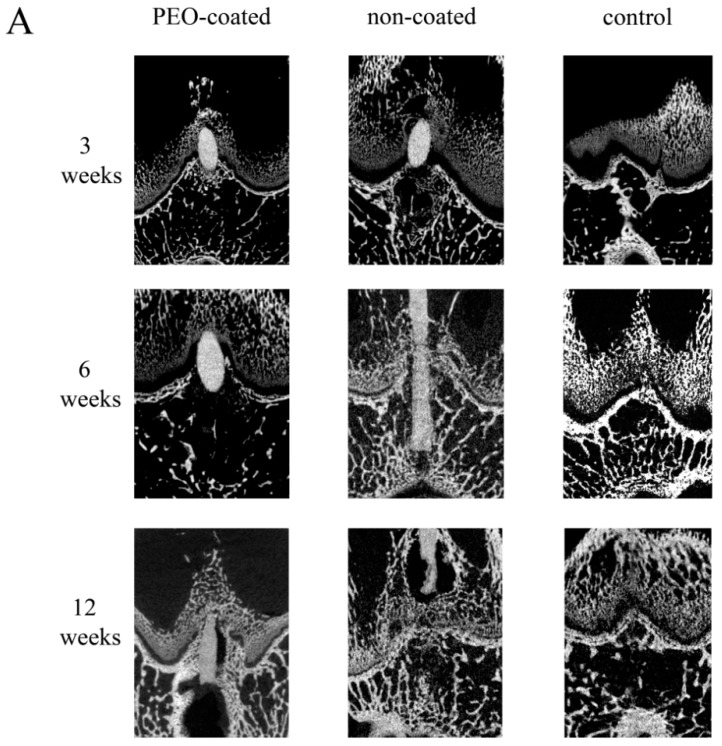

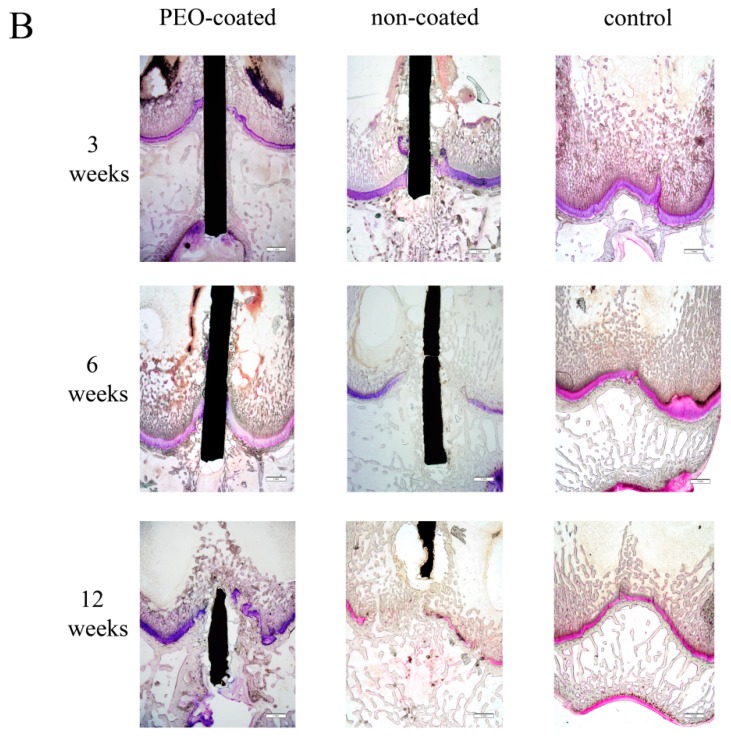
Micro-CT images (**A**) and histologic appearances (**B**) throughout the follow-up period. Note the scale bars represent 1 mm.

Both PEO-coated and non-coated Mg-Ca-Zn alloy pins began to degrade, even at three weeks. However, the two groups exhibited different tissue reactions (Figure 2). 

**Figure 3:**
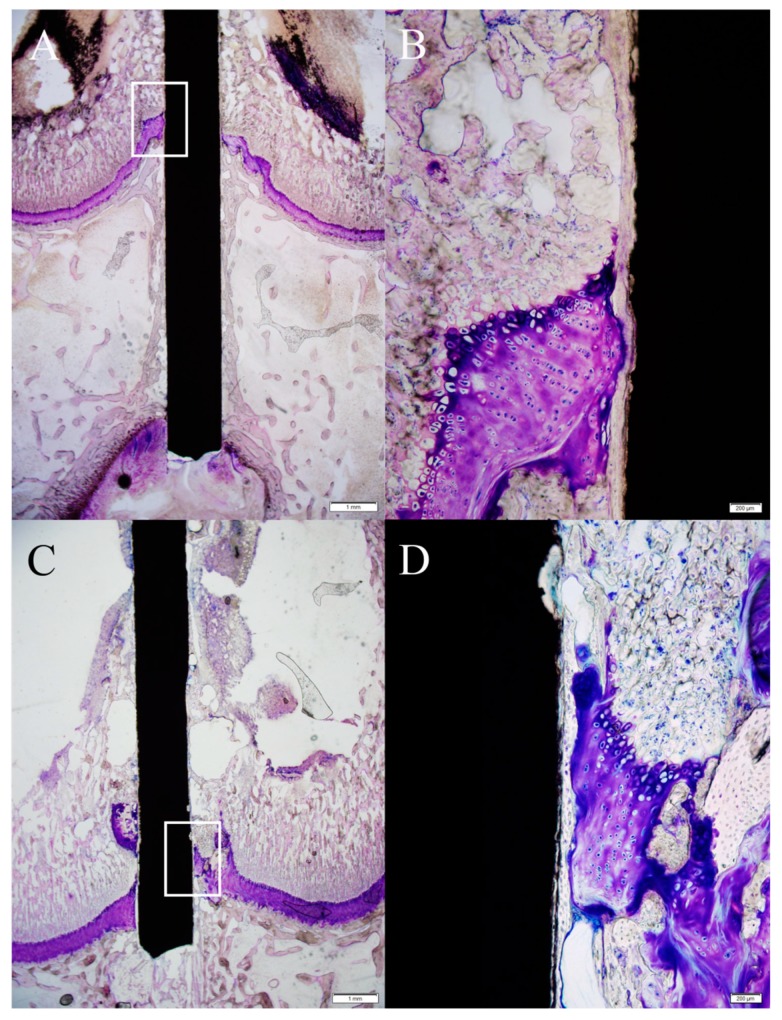
(**A**) In the PEO-coated Mg-Ca-Zn alloy pin, there was rare gas formation (scale bar: 1 mm). (**B**) Magnification of Figure 3A. The pin had an irregular surface, but tight bone integration formed over the pin surface. In addition, the growth plate adjacent to the pin showed mild hypertrophy and maintained orderly chondrocyte arrangement (scale bar: 200 μm). (**C**) In the non-coated Mg-Ca-Zn alloy pin, a large amount of gas formed (scale bar: 1 mm). (**D**) Magnification of Figure 3B. The pin surface was severely degraded compared to that of the PEO-coated group. The growth plate against the pin had orderly chondrocyte arrangement. Thickness of bone integration was shallower than that in the PEO-coated group (scale bar: 200 μm).

At three weeks, in the PEO-coated group (Figure 3A,B), the pin had an irregular surface and tight bone integration over the surface. There was rare gas formation. There were a few large black-colored granules with metallic debris adjacent to the tissues surrounding the Mg-Ca-Zn alloy pin. In addition, the growth plate at the pin insertion site had minute hypertrophy—however, it retained its orderly chondrocyte orientation. In contrast, in the non-coated group (Figure 3C,D), the pin had relatively severe surface degradation as compared to that in the PEO-coated group. Although there was bone integration, the thickness of the bone integration was shallower than that in the PEO-coated group. A large amount of gas was also noticed—in one specimen, hydrogen gas had formed across the growth plate during degradation. Large-sized black-colored granules with metallic debris were generated in the tissues adjacent to the Mg-Ca-Zn alloy pin. 

At six weeks, the degradation advanced in both PEO-coated and non-coated groups (Figure 2). The degradation in the non-coated group was relatively more severe than that in the PEO-coated group. The pins of the non-coated group were even fractured at the mid-point in four out of six specimens. In contrast, gas formation in the non-coated group was relatively greater than that in the PEO-coated group. A bone bridge has begun to appear in the process of degradation (Figure 2). 

At 12 weeks, the degradation was more advanced in both PEO-coated and non-coated groups than those three and six weeks after implantation (Figure 2). However, the status of the pin degradation was more aggressive in the non-coated group than it was in the PEO-coated group. Even the pins at the epiphysis and physis were almost entirely degraded, leaving only a trace of metallic debris in three out of six specimens of the non-coated group (Figure 2B). The bone bridge was observed in most of the specimens in both the PEO-coated and non-coated groups (Table 2). However, the bone bridge in the non-coated group was relatively larger than that in the PEO-coated group. 

### 2.3. Influence of the Mg-Ca-Zn Alloy Pin on the Growth Plate 

Bone bridge formation was observed in 13 of 36 specimens throughout follow-up—the occurrence and size of bone bridge (total area ratio) in each specimen are shown in Table 2. The bone bridge of the PEO-coated group did not exceed the inserted-pin diameter. In contrast, in the non-coated group, a large-sized bone bridge (much more exceeding pin diameter) formed. In addition, five specimens in the non-coated group developed premature physeal arrest (Figure 2B), which indicated almost complete closure of the growth plate (similar to that of mature bone). 

**Figure 4:**
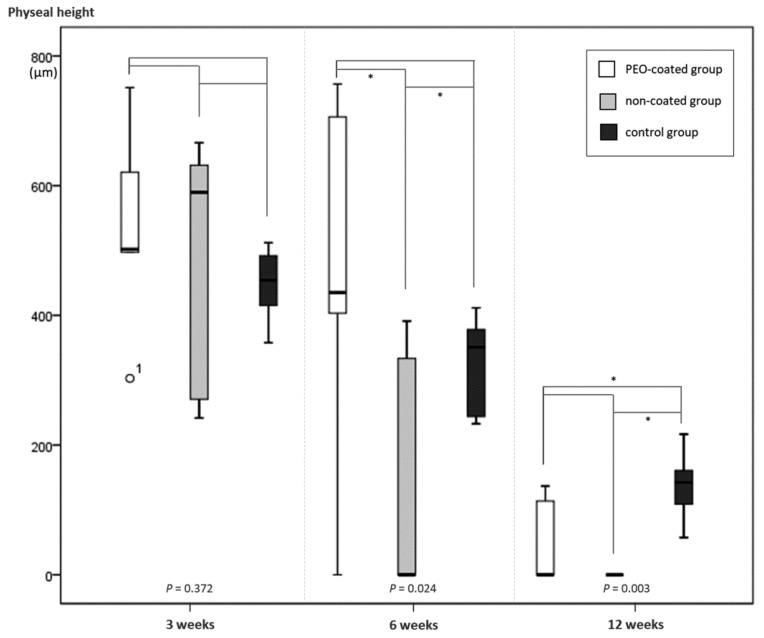
At 3 weeks, there was no significant difference in physeal height among groups (*p* = 0.372). At 6 weeks, physeal heights differed significantly among groups (*p* = 0.024). Physeal height in the non-coated group was lower than that in the other groups. At 12 weeks, physeal heights of both the PEO-coated and non-coated groups were lower than that of the control group (*p* = 0.003). Note the asterisk (*) means that the p-value was under 0.05.

The physeal heights, adjacent to the Mg-Ca-Zn alloy pin, decreased gradually during the follow-up period (Figure 4). At three weeks, the physeal heights did not differ significantly among the groups (*p* = 0.372). However, the growth plates in both the PEO-coated and non-coated groups seemed to be minutely hypertrophied as compared to that of the control group. At six weeks, the physeal heights differed significantly across groups (*p* = 0.024). The non-coated group had a noticeably narrower physeal height than did the others (*p* = 0.026 and 0.026, respectively). At 12 weeks, the physeal height of the PEO-coated group was as low as that of the non-coated group. The physeal heights of both PEO-coated and non-coated groups were lower than that of the control group (*p* = 0.026 and 0.002, respectively). 

Histopathologic analyses including chondrocyte orientation, density, and cell cloning are in Table 2. In addition, lymphocytes, plasma cells, and histiocytes infiltrations were not present in these specimens. These inflammatory cell infiltrates are characteristic features of a chronic inflammatory reaction. There was also no evidence of acute inflammatory reaction, which is characterized by neutrophil infiltration. Furthermore, there was no definite fibrosis. 

### 2.4. Femoral Segment Length 

**Figure 5:**
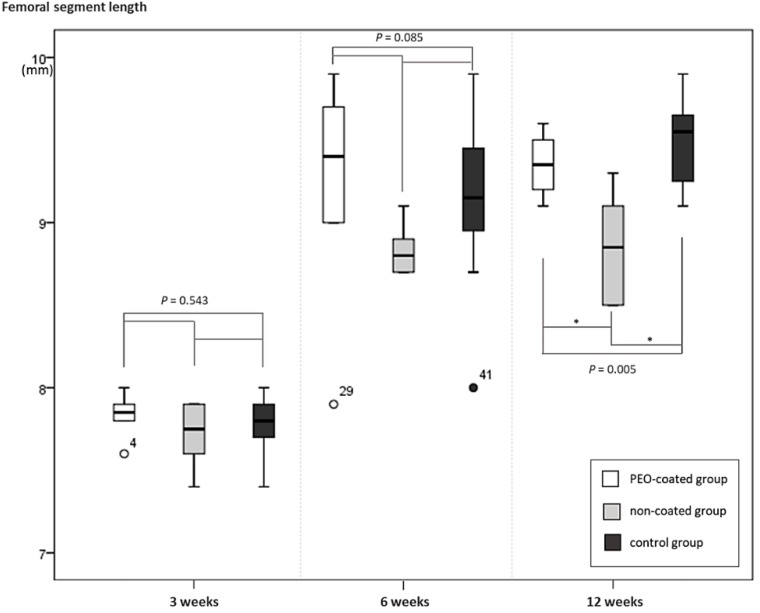
Until 6 weeks, there were no significant differences in femoral length among groups (*p* = 0.543 and 0.085, respectively). At 12 weeks, femoral segment lengths of the non-coated group were shorter than both the PEO-coated and control groups. Note the asterisk (*) represents that the *p*-value was under 0.05.

Femoral segment length throughout the follow-up period was shown in Figure 5. Until 6 weeks, there were no significant differences in femoral length among groups (*p* = 0.543 and 0.085, respectively). At 12 weeks, significant difference in femoral segment length developed among groups (*p* = 0.005). Femoral segment lengths of the non-coated group were shorter than both the PEO-coated and control groups. 

### 2.5. The Relationship between the Measurement Parameters 

**Figure 6:**
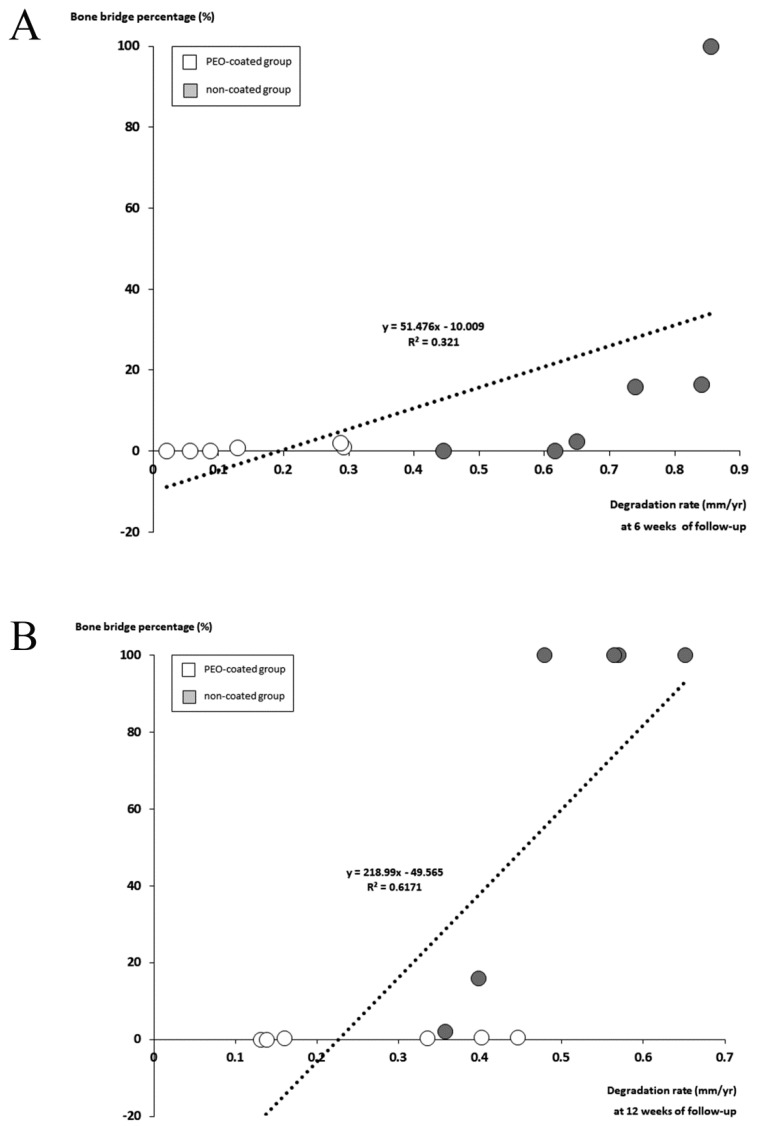
The relationship between the degradation rate and the size of bone bridge (total area ratio). (**A**) At the 6-week follow-up, the faster the Mg-Ca-Zn alloy pin degraded, the larger the bone bridge developed (OR, 2.323; 95% CI, 2.675 to 98.783; *p* = 0.04). (**B**) At the 12-week follow-up, the size of the bone bridge also closely depended on the degradation rate of the Mg-Ca-Zn alloy pin (OR, 4.014; 95% CI, 97.437 to 340.533; *p* = 0.002).

**Figure 7:**
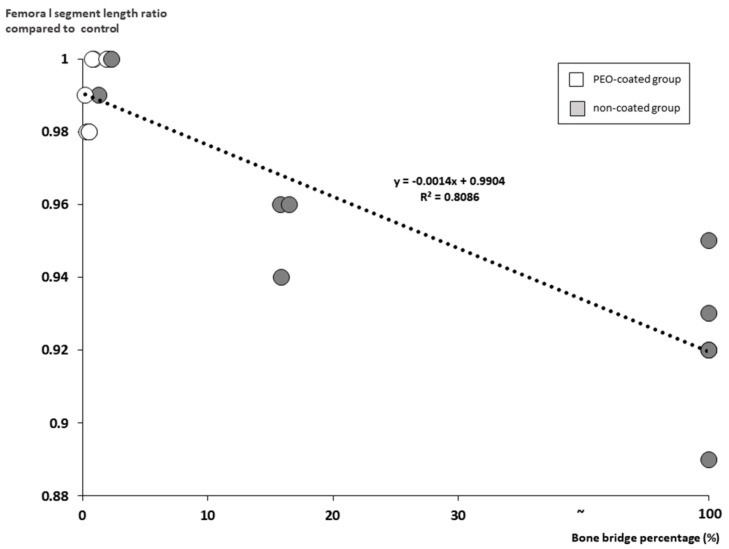
The large bone bridge significantly contributed to femoral shortening, which meant growth disturbance at the distal femoral physis (OR, −8.223; 95% CI, −0.002 to −0.001; *p* < 0.001).

The size of the physeal discontinuity (mostly bone bridge) closely depended upon the degradation rate of the Mg-Ca-Zn alloy pin (OR, 2.323; 95% CI, 2.675 to 98.783; *p* = 0.04 at six weeks of follow-up and OR, 4.014; 95% CI, 97.437 to 340.533; *p* = 0.002 at 12 weeks of follow-up) (Figure 6). The faster the pin degraded, the larger was the bone bridge developed. In addition, a large-sized physeal discontinuity (bone bridge) significantly contributed to the shortening of the femoral length in the process of Mg-Ca-Zn pin degradation (OR, −8.223; 95% CI, −0.002 to −0.001; *p* < 0.001) (Figure 7). 

## 3. Discussion

After inserting either PEO-coated or non-coated Mg-Ca-Zn alloy pins at the distal femoral physis, we examined the degradation performance of the Mg-Ca-Zn alloys and investigated their in vivo effects on the growth plate in a rabbit model. There were no acute or chronic inflammatory reactions in either PEO-coated or non-coated group. However, the two groups differed significantly in the tissue reactions to the degradation performance and physeal responses.

The current study used a newly developed Mg-Ca-Zn alloy. Prior study revealed its biocompatibility [18]: in histopathological analyses, acute or chronic inflammatory cells were rarely observed associated with a Mg-Ca-Zn alloy insertion. The degradation rate and mechanical properties of this Mg-Ca-Zn alloy was also investigated in another study [11]. In comparison with the other manufactured Mg alloys (AZ31, A91D, WE43, LAE442, and Mg-1CA), this Mg-Ca-Zn alloy had excellent degradation behavior (Table 3). Moreover, tolerable mechanical properties were identified (density: 1.80 mg/mm^3^, compressive strength: 415 MPa, tensile strength: 249 MPa). In addition, load bearing capacities including compression, tension, and bending were acceptable during the degradation performance.

The PEO-coating process used in this study was a surface modification method that originated from a traditional anodizing process. In this method, an oxide film is produced which then adheres to the Mg alloy surface during plasma discharge [20,21]. PEO-coating is known to be highly resistant to corrosion and wear on Mg alloys [8,20]. Concurrent with previous studies [22,23,24], we observed that PEO-coated Mg-Ca-Zn alloys degraded significantly more slowly and homogenously than did non-coated alloys. Noticeably less hydrogen gas also formed in the PEO-coated alloys than in the non-coated alloys during follow-up. Furthermore, PEO enhances the surface mechanical properties of Mg alloys and lengthens its stability as an implant [25]. We also observed in this study that relatively fewer cracks or fractures developed in the PEO-coated alloy pins that in the non-coated ones. In addition, PEO-coated alloys integrated with the medullary bone more tightly during degradation than non-coated alloys did.

On the growth plate, (1) the PEO-coated Mg-Ca-Zn alloys did not induce formation of a large bone bridge (that exceeds the inserted-pin diameter), whereas the non-coated Mg-Ca-Zn alloys tended to cause relatively large-sized bone bridges. Bone bridge formation may lead to the secondary tethering effect on the growth plate; (2) physeal height in the PEO-coated alloys was maintained and decreased more slowly than did that of the non-coated alloys. In contrast, there was a significant decrease in physeal height after six weeks of follow-up in the non-coated alloys. In addition, five specimens from the non-coated group experienced almost physeal closure, as in a mature bone. Thus, we did not observe significant growth disturbance on the growth plate associated with PEO-coated Mg-Ca-Zn alloys. However, non-coated Mg-Ca-Zn alloys significantly disturbed the growth in the distal femoral physis.

The different tissue reactions, including physeal responses, observed between the PEO-coated and non-coated alloys may depend on their degradation rate. When the interposition graft inappropriately dislocates from the resection cavity after physeal bar resection, the empty cavity is filled with hematoma, and the bone healing process begins [26]. This process leads to a recurrence in bone bridge formation and secondary tethering on the growth plate [27]. Similarly, inappropriately rapid degradation of the Mg-Ca-Zn alloy pin, especially in the non-coated group, could create a large empty space in the growth plate leading to the initiation of the process of bone bridge formation. Therefore, to decrease the risk of iatrogenic injuries due to Mg-Ca-Zn alloy insertion, the degradation rate must be slowed down. In this study, we found that the faster the pin degraded, the larger was the bone bridge developed. A bone bridge clearly appeared if the degradation rate was faster than 0.7 mm/year at the initial six weeks of follow-up and 0.4 mm/year at the 12 weeks of follow-up.

The mechanical tethering effect caused by the Mg-Ca-Zn alloy itself may also induce the growth disturbance in the distal femoral physis. However, this was not clearly observed in this study. In concurrence with a previous report [15], the biodegradable Mg-Ca-Zn pin began to degrade from the epiphysis and physis. We supposed that, as the Mg-Ca-Zn alloys degraded, the size of remaining Mg-Ca-Zn alloys across the physis was not large enough to produce the secondary tether effect on the growth plate.

As a result, large bone bridges contribute significantly to femoral length shortening during Mg-Ca-Zn pin degradation. We observed that bone bridges significantly disturbed growth if they made up >15% of the cross-sectional area of the growth plate. This result is consistent with previous reports, which noted that bone bridges that comprised >7% of the growth plate disturbed growth and shortened the femur [28,29].

Metallic debris, including Mg^2+^ ions, was produced during implant degradation. The metallic debris included large-sized, black-colored granules that were released into the surrounding tissues. The granules tended to be too large to enter the blood vessels and move to distant tissues. A prior study investigated the concentrations of Mg^2+^, Ca^2+^, and Zn^2+^ in local tissues during the degradation of Mg-Ca-Zn alloys [18]. They demonstrated that the concentration levels of these metallic elements did not significantly exceed their normal ranges within the natural tissue. Therefore, if the Mg-Ca-Zn alloy degradation rate was sufficiently low, the concentration levels of Mg, Ca, and Zn would be maintained within their normal ranges. Metallic debris was observed more commonly with the non-coated Mg-Ca-Zn alloys than with the PEO-coated alloys throughout the follow-up period. However, we did not quantify this observation.

The limitation of the current study was that it was conducted for only 12 weeks using skeletally immature New Zealand white rabbits (six weeks of age). This time point, a 12-week follow-up, was selected because the femoral segment of rabbits reaches 95% of adult length at 16 weeks (on average) [30]. However, humans have a much longer growth period than rabbits, reaching skeletal maturity at 16 years. We were not able to investigate the potential long-term adverse effects of Mg-Ca-Zn alloys on the growth plate. In addition, we only focused on Mg-Ca-Zn alloys without comparative analyses of other various Mg alloys. The reason is that the authors preferred Ca and Zn in composite materials and were reluctant to use either aluminum or heavy rare earth elements due to their toxic effects [10,15,16,17]. Although the only previous study that investigated the effect of biodegradable Mg alloys on the growth plate used yttrium in their Mg alloy (ZX50) [15], this is the first study to evaluate the in vivo effects of Mg alloys not containing potentially toxic substances on the growth plate. We believe that our study constitutes great clinical significance because this alloy only contained biocompatible elements. Based on the results of this study, the potential to development as various implants of Mg-Ca-Zn alloys, especially PEO-coated Mg-Ca-Zn alloys, could be verified.

## 4. Materials and Methods

### 4.1. Alloys

Two different types of Mg-Ca-Zn alloy pins (U&I Corporation, Gyeonggi-do, Korea) were used. The chemical compositions of the pins were identical and are as follows: 94 wt% Mg, 5 wt% Ca, and 1 wt% Zn. However, the surface modifications differed between the pins. One was a PEO-coated pin, which was coated with an oxide layer at the metal-electrolyte interface by a plasma discharge above the critical value [8,20]. In contrast, the other was not coated. Each pin was 1.3 mm in diameter and 35 mm long with a bayonet type tip.

### 4.2. Experimental Animals and Overall Management

This study was approved by the Institutional Animal Care and Use Committee (IACUC No. 15-0140-S1A0; Approved on July 3, 2015; Supplementary Materials). All animal experiments were conducted in accordance with the National Institutes of Health guide for the care and use of Laboratory animals (NIH Publications No. 8023, revised 1978).

**Figure 8:**
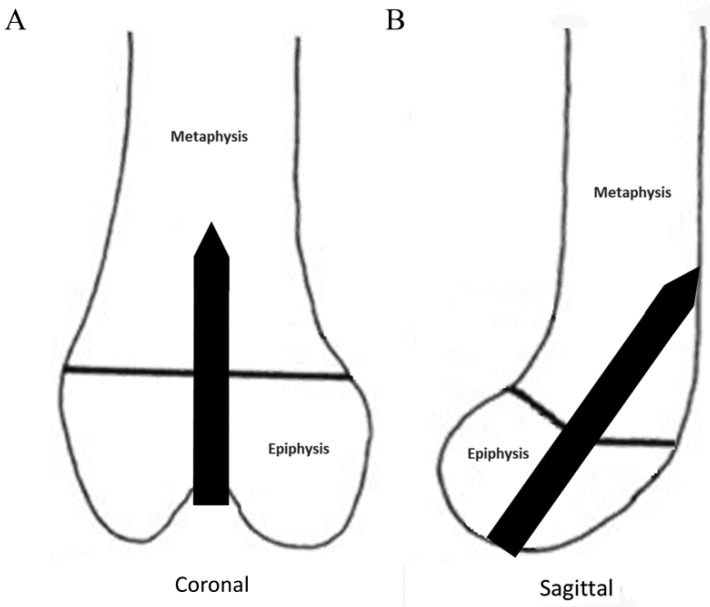
Insertion method of a Mg-Ca-Zn alloy pin. (**A**) In the coronal plane, a pin was inserted in the center of the femoral intercondylar notch perpendicular to the growth plate. (**B**) In the sagittal plane, a pin was inserted into the distal femoral anterior cortex through the center of the physis.

Thirty-six New Zealand white rabbits at six weeks of age and weight 800–1200g (Coatech Co., Gyeonggi-do, Korea) were used. The rabbits were anesthetized with an intravenous injection of zoletil (10 mg/kg, Virbac SA, Carros, France) and xylazine (20 mg/kg, Bayer Korea, Seoul, Korea). After shaving and sterile preparation, a medial parapatellar incision was made. The patella was dislocated laterally to expose the knee joint. Under fluoroscopic guidance, a Mg-Ca-Zn alloy pin (1.3-mm diameter; approximately 1%–2% relative to the entire cross-sectional area of the distal femoral growth plate) was inserted from the femoral intercondylar notch to the distal femoral anterior cortex through the center of the physis (Figure 8). Half of the rabbits (*n* = 18) underwent PEO-coated pin fixation, while the other half underwent non-coated pin fixation. The contralateral left side was operated in the same manner, but the pin was removed immediately. After the operation, the patella was repositioned, and the wound was closed in layers with 2-0 Ethilon sutures. The rabbits were treated with intravenous cefazolin (30 mg/kg, Apothecon, Bristol-Myers Squibb, Princeton, NJ, USA) and ketorolac at 1 mg/kg postoperatively. The rabbits were housed in individual cages with no restriction on diet or exercise.

Six rabbits from each group were euthanized at each of the three-, six-, and twelve-week follow-ups by intravenously injecting anesthetics and 0.3 mL of KCl (JW Pharmaceutical Co., Seoul, Korea). Seventy-two femoral bone specimens were obtained.

### 4.3. Micro-CT Analyses

All of the specimens were analyzed using a micro-CT scanner (Skyscan 1076; SkyScan, Aartselaar, Belgium). The RV of the pin and void volume were measured at six and 12-week follow-ups, respectively, using a Micro-CT Analysis Tool (programmed by PhD. Sang Joon Park, Seoul, Korea). The degradation percentage and rate were also calculated. The degradation rate was derived as follows [11]:

$$\mathrm{Degradation}\;\mathrm{rate}\;(\mathrm{mm}/\mathrm{year})\;=\;\frac{\mathrm{initial}\;\mathrm{volume}\;-\;\mathrm{RV}\;}{\;\mathrm{initial}\;\mathrm{surface}\;\mathrm{area}\;x\;\mathrm{time}}$$

where initial volume was 44.87 mm^3^ and initial surface area was 141.83 mm^2^.

In addition, the effects of the Mg-Ca-Zn alloy pins and their by-products on the growth plate were investigated. We recorded the development of physeal discontinuity related to the degradation of the Mg-Ca-Zn alloy pin, and the percentage of it to the entire growth plate. Premature physeal arrest was also investigated and defined by the almost complete disappearance of the physeal cartilage (except a trace of it).

The femoral segment length was also measured after three, six, and twelve-week follow-ups with the RadiAnt DICOM Viewer (CT viewer program, SkyScan). The femoral segment length was defined as the distance from the top of the femoral head to the distal end of the medial epicondyle.

### 4.4. Histologic Analyses

All specimens were fixed in 99.5% ethyl alcohol for tissue dehydration. The specimens were infiltrated with the embedding media (Technovit 7200 VLC; Exakt, Hamburg, Germany) and polymerized. Each specimen was grounded and polished down to tissue sections of 5 µm in thickness using a micro-grinding system (Exakt). Undecalcified bone sections were stained with modified hematoxylin and eosin staining (Polyscience Inc., Warrington, PA, USA).

The overall histologic appearance during the degradation of the PEO-coated and non-coated Mg-Ca-Zn alloy pins were investigated at weeks 3, 6, and 12 after implantation.

We also performed histomorphometric and histopathologic analyses of the growth plate. The entire growth plate thickness (physeal height), including resting, proliferative, and hypertrophic zones, was measured. The width of the physeal discontinuity (related to degradation of Mg-Ca-Zn alloy pin) was measured, and the interposed substances were investigated. A growth plate was considered to be in premature physeal arrest if it almost completely disappeared, leaving only a trail of chondroid matrix like a mature bone. Chondrocyte orientation, density, and cell cloning were also assessed according to the Osteoarthritis Research Society International Recommendations [19]. The presence of any Mg-Ca-Zn alloy pin-related inflammatory reaction of the physis was also evaluated.

### 4.5. Statistical Analysis

Numerical variables that did not follow a normal distribution were analyzed with the Wilcoxon signed-rank test and the Mann-Whitney test for paired and unpaired data, respectively. Numerical variables that followed a normal distribution were analyzed with the paired t-test and the independent t-test for paired and unpaired data, respectively. Numerical variables were compared among the three groups with the Kruskal-Wallis test. We then performed univariate logistic regression analysis to determine the relationship between the degradation rate and the size of physeal discontinuity (or defect). Univariate logistic regression analysis was also used to determine the relationship between the size of the physeal discontinuity and the growth disturbance of the physis. Statistical analyses were performed with SPSS, version 21.0 (SPSS, Inc., Chicago, IL, USA). Finally, *p* values < 0.05 were considered statistically significant.

## 5. Conclusions

Although there were no inflammatory reactions in both PEO-coated and non-coated Mg-Ca-Zn alloys, the PEO-coated and non-coated Mg-Ca-Zn alloys had significantly different responses on the growth plate. Depending on degradation rate, large bone bridge formation and premature physeal arrest mainly occurred in the non-coated group which exhibited rapid degradation; however, only a small-sized bone bridge formed in the PEO-coated group, which degraded at a slower rate compared to the non-coated group. This difference ultimately led to a significant discrepancy of the femoral segment length between the groups. Our study suggests that PEO-coated Mg-Ca-Zn alloys can be a useful material for achieving optimal degradation to avoid damage to growth plate, thus projecting these alloys as a promising and safe biodegradable material in pediatric orthopaedics. Nevertheless, further research is needed to substantiate these findings prior to clinical application.

## Figures and Tables

**Table 1:** Quantitative analysis of micro-CT imaging.

Follow-Up Period (Weeks)	6			12		
	PEO-Coated Group	Non-Coated Group	*p* Value **	PEO-Coated Group	Non-Coated Group	*p* Value **
No. of animals	6	6		6	6	
Residual volume (RV) (mm^3^)	42.5 (40.1 to 44.5) *	33.6 (30.9 to 37.6) *	0.010	36.1 (30.3 to 40.6) *	28.4 (23.5 to 33.2) *	0.026
Void volume (VV) (mm^3^)	2.4 (1.1 to 4.3) *	19.4 (13.9 to 34.2) *	0.010	19.4 (8.6 to 33.7) *	42.1 (15.5 to 67.3) *	0.132
Degradation percentage (%)	5.3 (0.8 to 10.6) *	25.2 (31.2 to 16.2) *	0.010	19.6 (0.6 to 32.5) *	36.7 (26.1 to 47.5) *	0.026
Degradation rate ^†^ (mm/year)	0.145 (0.021 to 0.292) *	0.690 (0.445 to 0.856) *	0.010	0.269 (0.131 to 0.446) *	0.503 (0.357 to 0.651) *	0.026

* Data in parenthesis represent ranges; ** Mann-Whitney test; ^†^ Degradation rate was calculated as follows [11]: degradation rate (mm/year) = $\frac{\mathrm{initial}\;\mathrm{volume}\;-\;\mathrm{RV}\;}{\;\mathrm{initial}\;\mathrm{surface}\;\mathrm{area}\;x\;\mathrm{time}}$; where initial volume is 44.87 mm3 and the initial surface area is 141.83 mm^2^.

**Table 2:** Summary of the effects of a Mg-Ca-Zn alloy pin on the growth plate.

Follow-Up Period (weeks)	Group	Rabbits	Physeal Height (µm)	Chondrocyte		Cell Cloning ^†^	Physeal Discontinuity *			Femoral Segment Length	
				Orientation	Density ^†^		Percentage (%)	Width (µm)	Interposed Substance	Length (mm) (Ipsilat./Contralat.)	Ratio Compared to Control
3	PEO-coated	1	302.8	orderly	1	2	-	-	-	78/78	1
		2	499.9	orderly	1	0	-	-	-	79/79	1
		3	751.2	orderly	3	4	-	-	-	79/79	1
		4	497.4	orderly	1	0	-	-	-	76/76	1
		5	504.3	orderly	0	1	-	-	-	80/80	1
		6	620.8	orderly	2	1	-	-	-	78/78	1
	Non-coated	1	549.4	orderly	1	4	-	-	-	78/78	1
		2	631.6	orderly	0	4	-	-	-	79/79	1
		3	666.1	orderly	1	0	-	-	-	74/74	1
		4	630.4	orderly	1	1	-	-	-	76/76	1
		5	303.3	orderly	1	4	1.3	492	Void area (Hydrogen gas)	77/78	0.99
		6	270.7	orderly	0	2	-	-	-	79/79	1
	Control	(average)	466.4	orderly	0 to 1	0 to 1	0.8	325.3	Bone bridge	-	-
6	PEO-coated	1	403.3	orderly	1	1	0.9	511	Bone bridge	96/96	1
		2	0	disorderly	2	0	2.0	733	Bone bridge	92/92	1
		3	428.7	orderly	1	0	0.8	250	Bone bridge	90/90	1
		4	706.1	orderly	1	0	-	-	-	99/99	1
		5	756.6	orderly	1	0	-	-	-	97/97	1
		6	441.5	orderly	3	0	-	-	-	79/80	0.99
	Non-coated	1	0	disorderly	4	4	15.8	3168	Bone bridge	88/92	0.96
		2	0	disorderly	4	4	16.5	3656	Bone bridge	87/91	0.96
		3	0	N/C	4	4	Premature physeal arrest			88/93	0.95
		4	0	disorderly	1	1	2.3	920	Bone bridge	89/89	1
		5	391.1	orderly	1	4	-	-	-	87/87	1
		6	333.6	disorderly	2	4	-	-	-	91/91	1
	Control	(average)	309.7	orderly	0 to 1	0 to 1	0.9	351.7	Bone bridge	-	-
12	PEO-coated	1	113.5	orderly	0	4	-	-	-	92/92	1
		2	0	disorderly	2	4	0.2	103	Bone bridge	95/96	0.99
		3	0	disorderly	2	4	0.3	267	Bone bridge	91/93	0.98
		4	0	disorderly	2	4	0.5	444	Bone bridge	94/96	0.98
		5	0	orderly	2	2	0.5	487	Bone bridge	96/98	0.98
		6	136.8	orderly	0	4	-	-	-	93/93	1
	Non-coated	1	0	N/C	4	4	Premature physeal arrest			85/96	0.89
		2	0	N/C	4	4	Premature physeal arrest			89/97	0.92
		3	0	N/C	4	4	Premature physeal arrest			85/92	0.92
		4	0	disorderly	3	1	1.8	467	Bone bridge	9.1/9.1	1
		5	0	disorderly	4	4	15.9	3487	Bone bridge	9.3/9.9	0.94
		6	0	N/C	4	4	Premature physeal arrest			8.8/9.5	0.93
	Control	(average)	154.1	orderly	0 to 1	0 to 1	0.9	330.5	Bone bridge	-	-

* Physeal discontinuity, except pin remnant, occurred in every growth plate. Premature physeal arrest was defined as the almost complete disappearance of physeal cartilage (except a trace of it in the micro-CT and histologic analyses); ^†^ Chondrocyte density and cell cloning adjacent to the insertion site were examined according to the method of the Osteoarthritis Research Society International [19].

**Table 3:** Comparison of degradation rate of various Mg alloys.

Materials	Degradation Rate (mm/yr) *
AZ31 ^†^	0.650
AZ91D ^†^	0.770
WE43 ^†^	0.868
LAE442 ^†^	0.218
Mg-1Ca ^†^	1.269
Mg-Ca-Zn (non-coated) ^†^	0.348 to 0.690
Mg-Ca-Zn (PEO-coated, current study)	0.145

Note the asterisk (*) means the value of average. ^†^ Data was cited from reference [11].

## Supplementary Materials

Supplementary materials can be found at https://www.mdpi.com/1422-0067/20/15/3761/s1.

## Abbreviations

RV	residual volume
PEO	plasma electrolyte oxidation
Ca	calcium
Zn	zinc
CT	computed tomography
