# Supplementary material for: In Vivo Response of Growth Plate to Biodegradable Mg-Ca-Zn Alloys Depending on the Surface Modification
Source: Int J Mol Sci. 2019 Aug 1;20(15):3761. doi: 10.3390/ijms20153761 (PMC6695941; doi:10.3390/ijms20153761)
Supplement: Supplementary file 1 [file ijms-20-03761-s001.zip › Supplementary file/1. State of Significance.docx]

**Statement of Significance**

A previous study demonstrated that biodegradable Mg alloys including yttrium did not adversely affect the growth plate, but they recommended not to use Mg alloys containing rare earth elements for preventing long-term toxic effects on adjacent and distant tissues. In contrast, in M-Ca-Zn alloys, all the components have good biocompatibility, and the corrosion resistance and mechanical properties are much improved compared to those of pure Mg.

This is the first study to evaluate the in vivo effects on the growth plate of Mg alloys that do not contain rare earth elements. Mg-Ca-Zn alloys have a slow degradation rate, making them a promising and safe biodegradable material that would not damage the growth plate. PEO coating may be an effective option to achieve optimal degradation of Mg-Ca-Zn alloys.
