# Supplementary material for: In Vivo Response of Growth Plate to Biodegradable Mg-Ca-Zn Alloys Depending on the Surface Modification
Source: Int J Mol Sci. 2019 Aug 1;20(15):3761. doi: 10.3390/ijms20153761 (PMC6695941; doi:10.3390/ijms20153761)
Supplement: Supplementary file 1 [file ijms-20-03761-s001.zip › Supplementary file/5. IRB Approval.pdf]

Institutional Animal Care and Use Protocol Approval

Name of Study	**Physeal Response to Biodegradable Mg-Ca-Zn Alloy Pins in Immature Rabbits**		
IACUC No.	15-0140	Study Period	Aug . 03 . 2015 . ~ Aug . 02 . 2016 .
Study No.	35 - 2015 - 009 - 6		

<Evaluation Criteria and Discussion Results>

**1**	The rationale and purpose of the proposed use of animals<br>V appropriate ☐ inappropriate	**7**	Unusual housing and husbandry requirements<br>☐ necessary V unnecessary
**2**	Justification of species and number of animals requested<br>V appropriate ☐ inappropriate	**8**	Appropriate sedation, analgesia, and anesthesia<br>V appropriate ☐ inappropriate
**3**	Unnecessary duplication of tests or experiments<br>V appropriate ☐ inappropriate	**9**	Method of euthanasia or disposition of animal<br>V appropriate ☐ inappropriate
**4**	Availability or appropriateness of the use of less-invasive procedures<br>V appropriate ☐ inappropriate	**10**	Criteria and process for timely intervention, removal, or euthanasia if required<br>V appropriate ☐ inappropriate
**5**	Adequacy of training and experience of personnel<br>V appropriate ☐ inappropriate	**11**	Safety of working environment for personnel<br>V appropriate ☐ inappropriate
**6**	Conduct of multiple major surgical procedures<br>V appropriate ☐ inappropriate	Remarks:	

Date of Submission July. 03, 2015
Principle Investigator TAE JOON CHO
Date of Approval July. 22, 2015
IACUC Chair Kook Hyun Lee (sign)

Seoul National University Hospital (Tel: +82-2-2072-1902, Fax: +82-2-3675-8335)
E-mail: iacuc@snuh.org http://cri.snuh.org

SNUH-IACUC
